# Supplementary material for: Soluble dimeric prion protein ligand activates Adgrg6 receptor but does not rescue early signs of demyelination in PrP-deficient mice
Source: PLoS One. 2020 Nov 12;15(11):e0242137. doi: 10.1371/journal.pone.0242137 (PMC7660510; doi:10.1371/journal.pone.0242137)
Supplement: S1 Fig — The uncropped images have been inverted and autoscaled using the Quantify One software (Biorad). Lanes with protein size markers (Protein Precision Plus, Biorad) are indicated with M. Relevant size markers are marked with the respective size in kD. The specific band is marked with * when additional non-specific bands are present. Irrelevant lanes that have been excluded from the main figures are marked with x. The samples that were loaded in these lines are described in annotations. Lanes that were left empty are marked with e. The samples loaded in all other lanes were specified in the main figures. (PDF) [file pone.0242137.s001.pdf]

Figure 1b

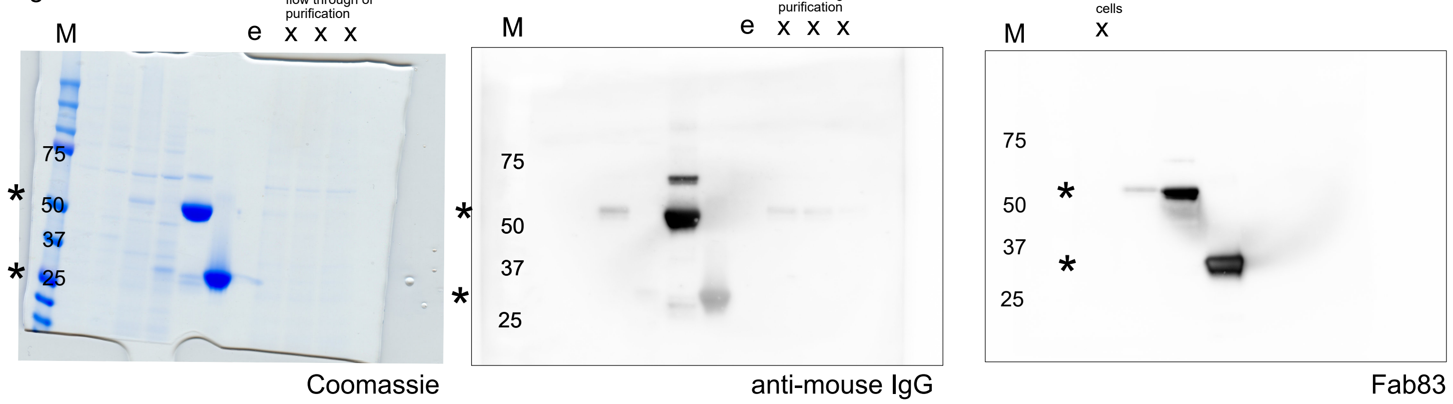

Figure 1d

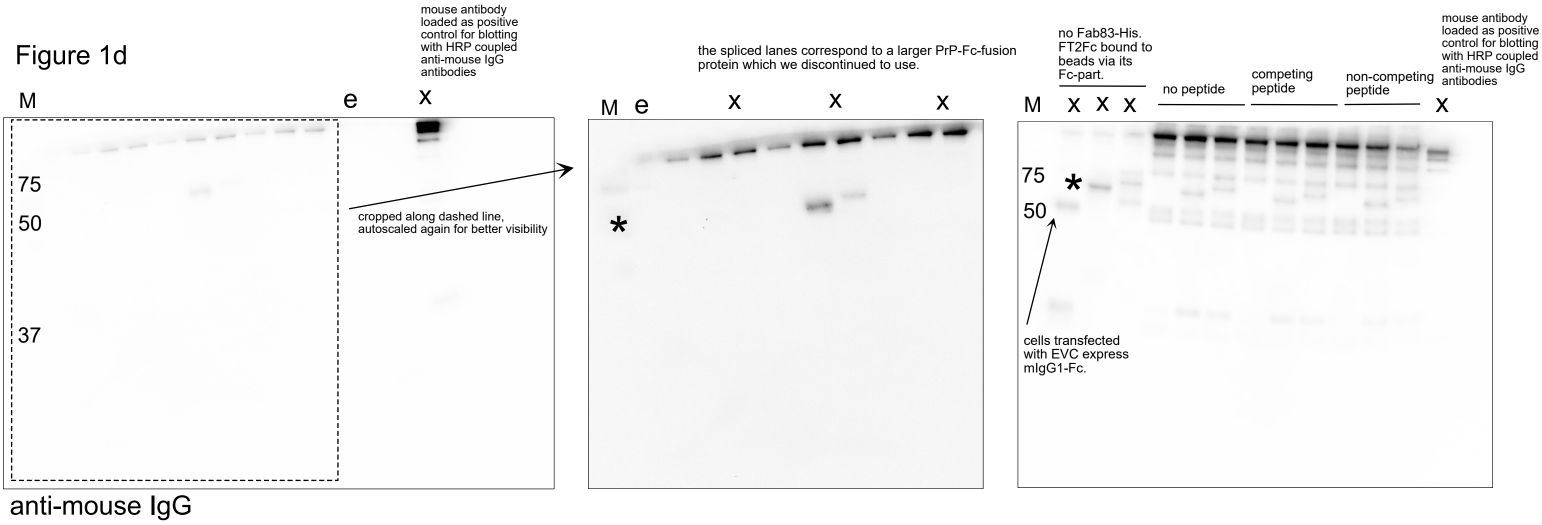

Figure 2c

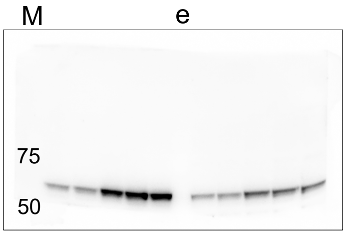

pAKT

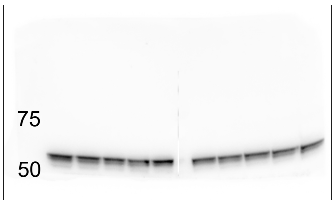

AKT

For pAKT and AKT staining here and in all following blots, pAKT staining was performed first, then the blot was stripped and incubated with antibodies against AKT.
The gel was cut below or above 50 kD prior to incubation with primary antibodies to allow for staining of other proteins of interest.

Figure 3a

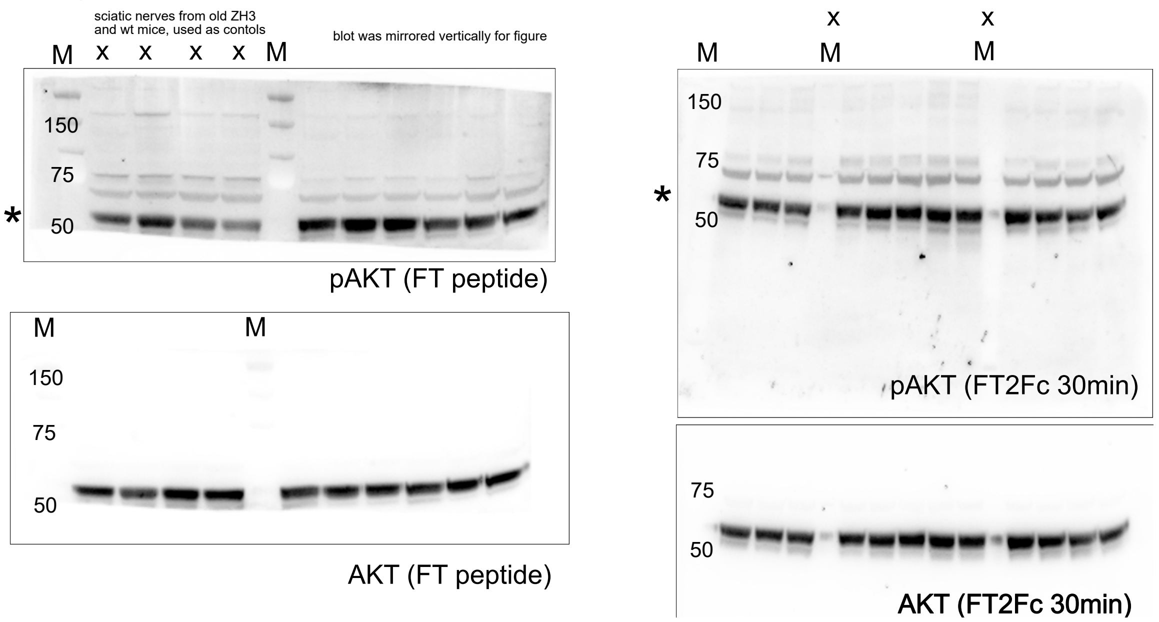

Figure 3b

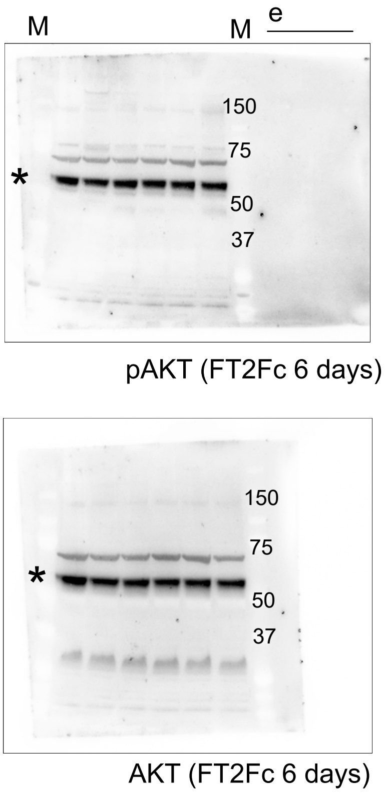

Figure 3c

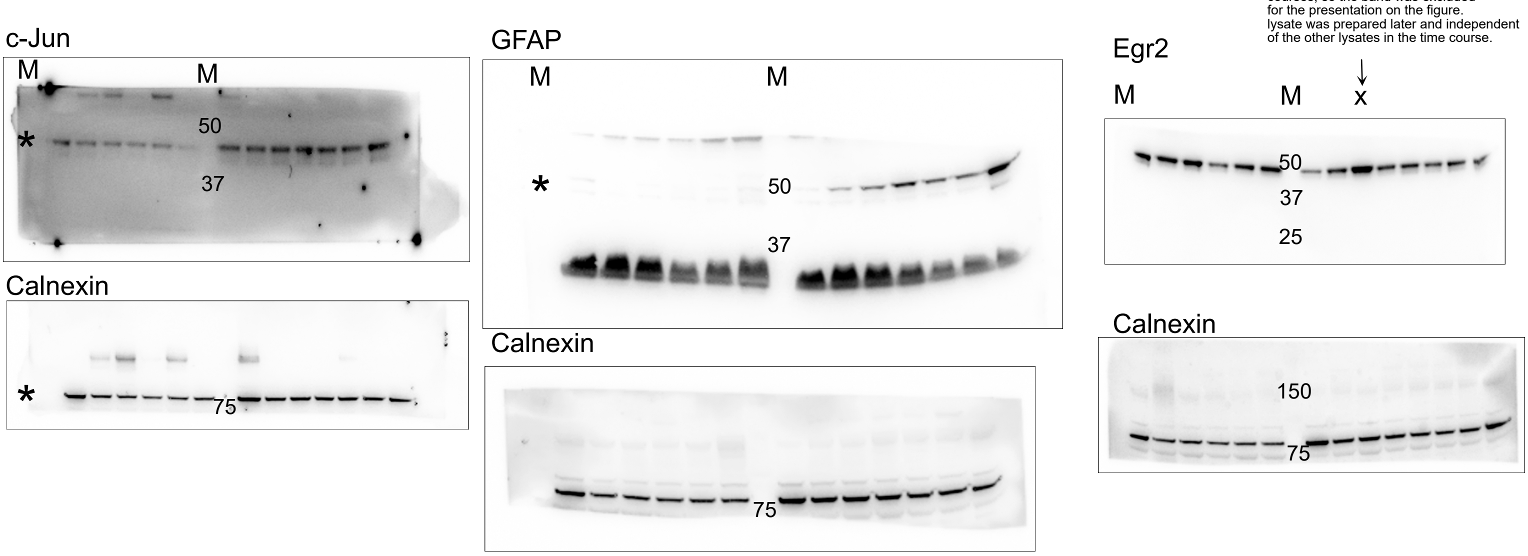

Figure 3d

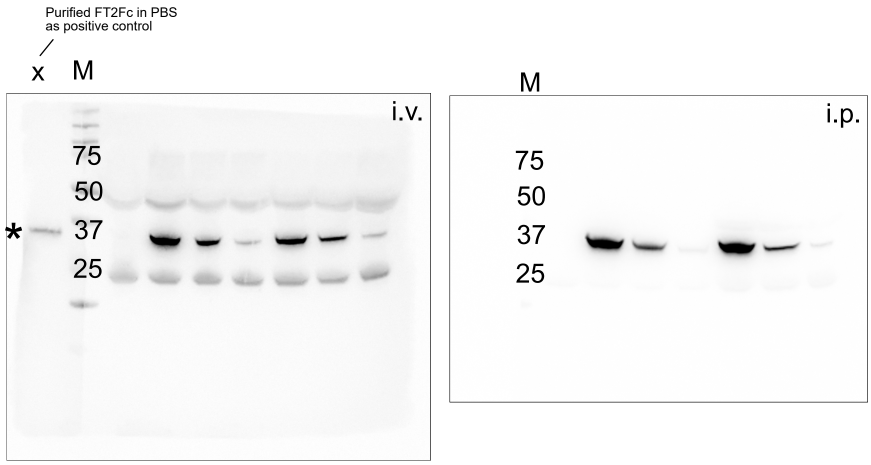

Figure 3e

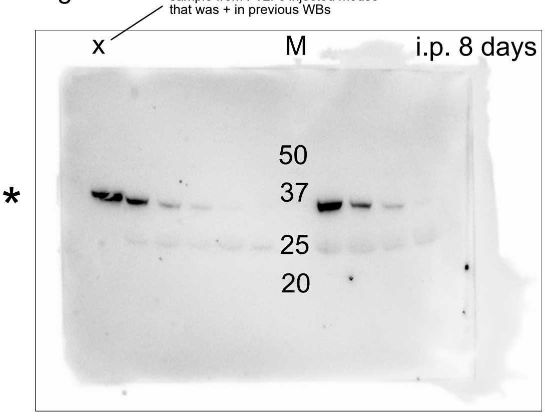

Figure 4b

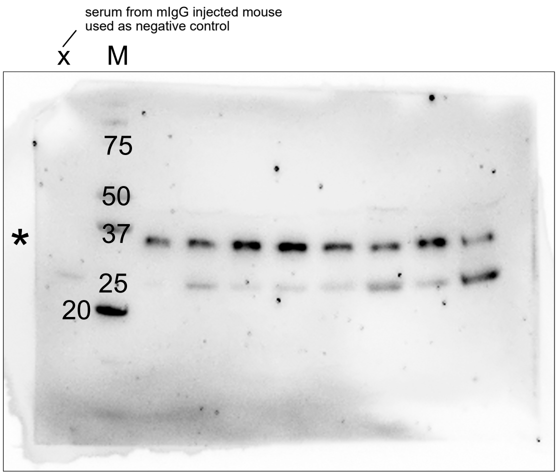

Figure 4d
Blots for Egr2 + Actin and c-Jun + Calnexin were mirrored vertically for presentation in figure.

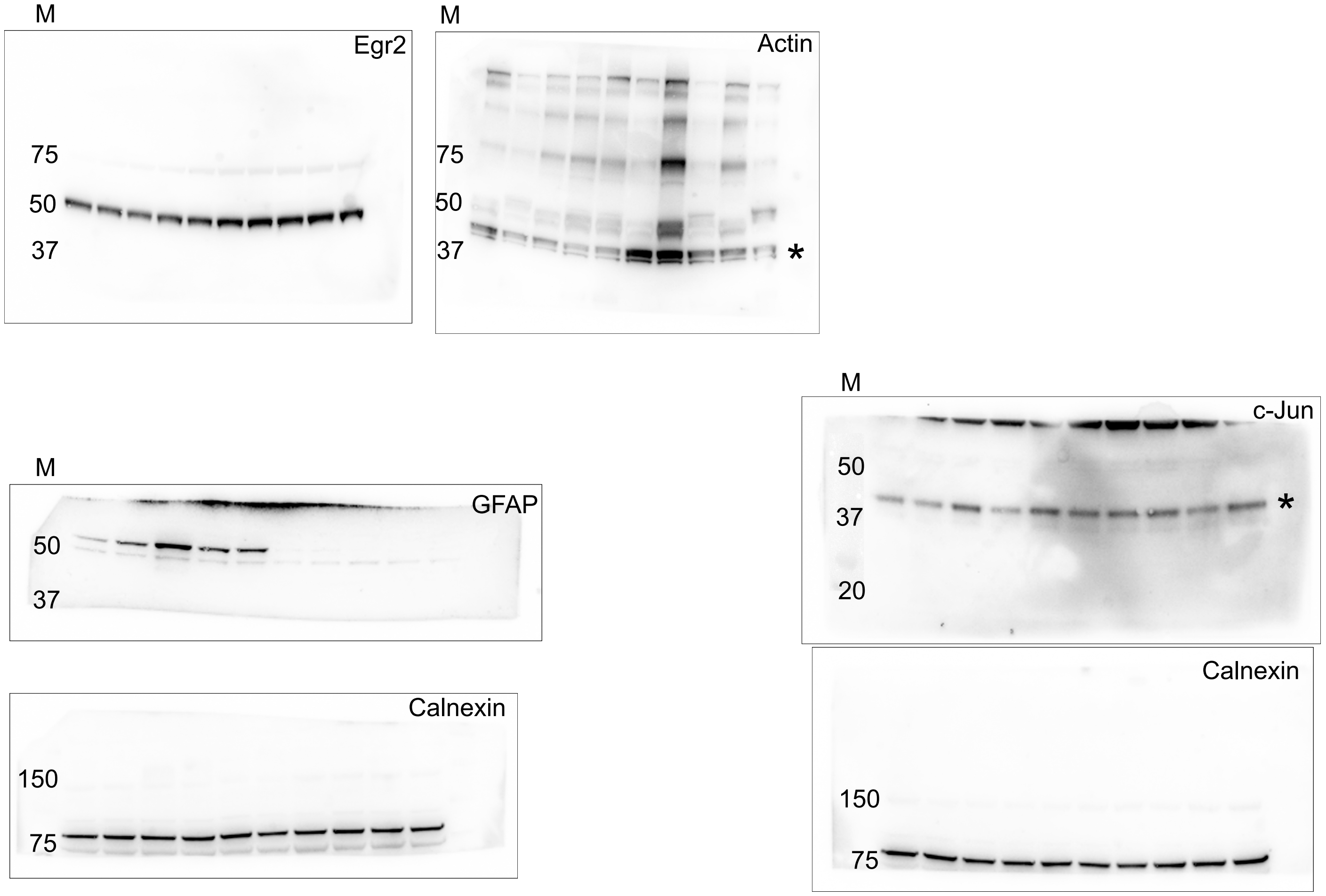

S2 Fig

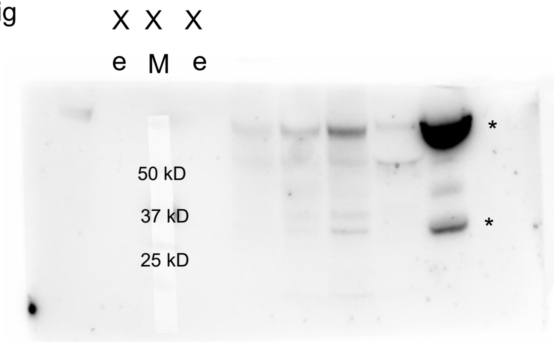

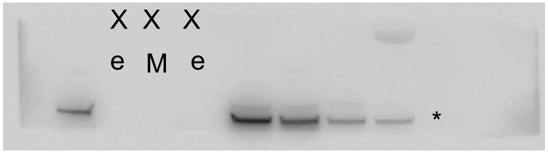

Calnexin
