# Supplementary figures and images for: Soluble dimeric prion protein ligand activates Adgrg6 receptor but does not rescue early signs of demyelination in PrP-deficient mice
Source: PLoS One. 2020 Nov 12;15(11):e0242137. doi: 10.1371/journal.pone.0242137 (PMC7660510; doi:10.1371/journal.pone.0242137)

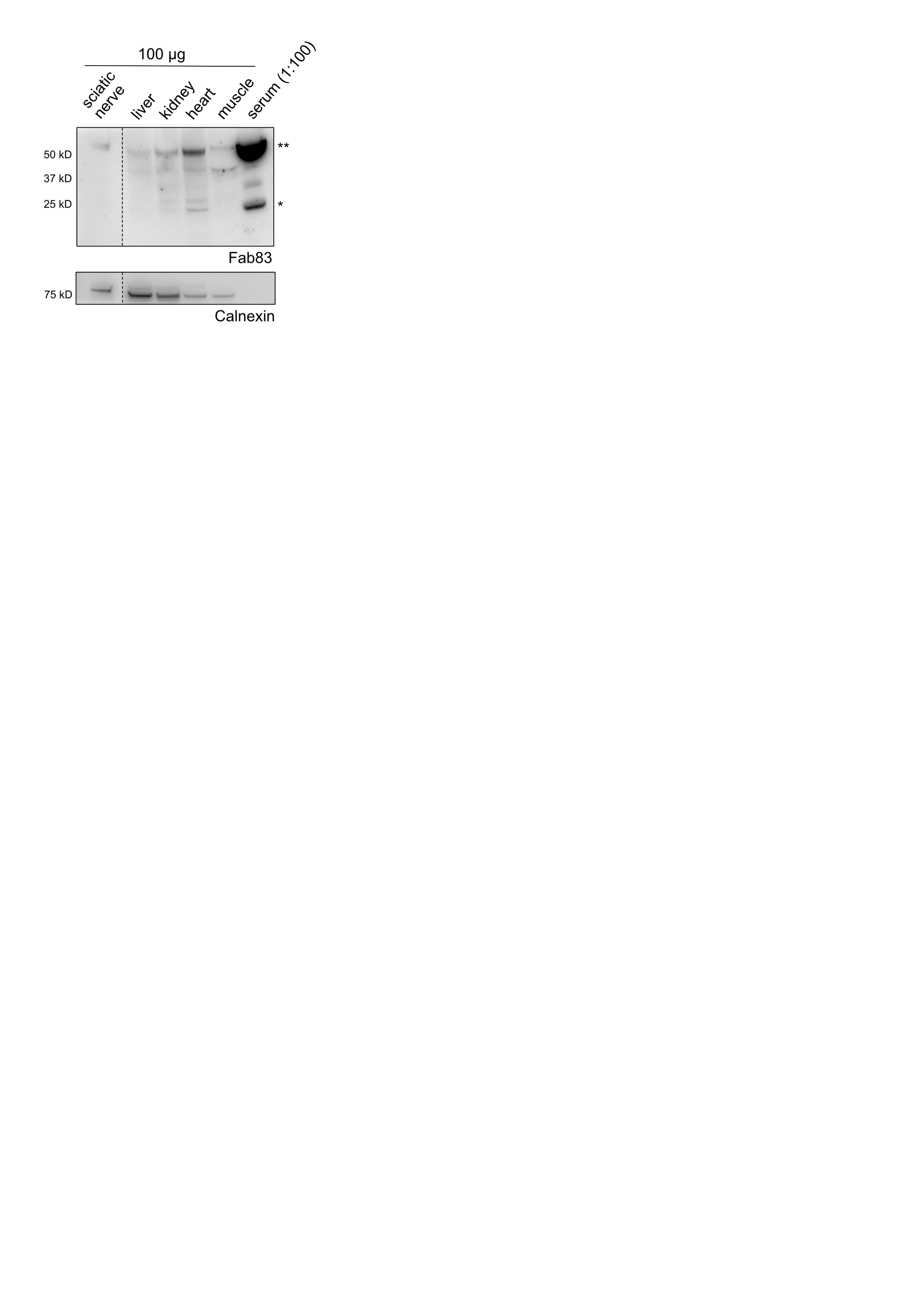

Supplement: S2 Fig — Tissue lysates from a mouse chronically treated with FT2Fc were subjected to western blotting with Fab83. FT2Fc was detected in all organs (100 μg of total protein) and in the serum (diluted 1:100 in PBS). Calnexin was used as a loading control. The mouse was sacrificed 2 days after the last injection. **: dimer. *: monomer. (TIF) [file pone.0242137.s002.tif]
