# Supplementary material for: Soluble dimeric prion protein ligand activates Adgrg6 receptor but does not rescue early signs of demyelination in PrP-deficient mice
Source: PLoS One. 2020 Nov 12;15(11):e0242137. doi: 10.1371/journal.pone.0242137 (PMC7660510; doi:10.1371/journal.pone.0242137)
Supplement: S1 Table — (DOCX) [file pone.0242137.s003.docx]

*Supplementary Table 1*: Complete list of overrepresented GO categories in sciatic nerves of FT_2_Fc treated compared to buffer treated mice.

**Enriched categories among downregulated genes**

| **Biological Process (BP) category** | **ID** | **p-value**  **(adjusted)** | **Number of genes** |
| --- | --- | --- | --- |
| striated muscle myosin thick filament assembly | GO:0071688 | 0.00e+00 | 6/7 |
| muscle contraction | GO:0006936 | 2.41e-26 | 18/40 |
| .regulation of muscle contraction | GO:0006937 | 1.15e-8 | 6/18 |
| .striated muscle contraction | GO:0006941 | 2.25e-15 | 10/18 |
| . .skeletal muscle contraction | GO:0003009 | 7.36e-8 | 6/20 |
| . .cardiac muscle contraction | GO:0060048 | 1.54e-7 | 7/38 |
| sarcomere organization | GO:0045214 | 1.69e-19 | 13/29 |
| actin filament organization | GO:0007015 | 1.53e-9 | 10/87 |
| .skeletal muscle thin filament assembly | GO:0030240 | 5.31e-7 | 4/6 |
| cardiac muscle tissue morphogenesis | GO:0055008 | 3.68e-7 | 5/13 |
| cardiac muscle fiber development | GO:0048739 | 5.26e-7 | 4/8 |
| glycogen metabolic process | GO:0005977 | 4.14e-06 | 5/34 |
| .glycogen catabolic process | GO:0005980 | 3.82e-06 | 3/7 |
| response to denervation involved in regulation of muscle adaptation | GO:0014894 | 2.29e-05 | 3/9 |
| skeletal muscle cell differentiation | GO:0035914 | 2.87e-05 | 5/40 |
| regulation of ion transmembrane transport | GO:0034765 | 4.37e-05 | 9/127 |
| adult heart development | GO:0007512 | 7.17e-05 | 3/10 |

| **Molecular Function (MF) category** |  |  |  |
| --- | --- | --- | --- |
| structural molecule activity conferring elasticity | GO:0097493 | 0.00e+00 | 6/8 |
| structural constituent of muscle | GO:0008307 | 4.77e-17 | 11/26 |
| structural constituent of cytoskeleton | GO:0005200 | 2.15e-11 | 10/60 |
| calmodulin binding | GO:0005516 | 1.89e-06 | 10/147 |
| voltage-gated ion channel activity | GO:0005244 | 3.86e-05 | 8/97 |
| cytoskeletal protein binding | GO:0008092 | 4.84e-05 | 5/57 |
| .actin binding | GO:0003779 | 4.91e-14 | 21/306 |
| . .actin filament binding | GO:0051015 | 2.31e-18 | 19/144 |
| .titin binding | GO:0031432 | 1.53e-8 | 5/8 |
| .FATZ binding | GO:0051373 | 1.58e-05 | 3/4 |

| **Cellular Component (CC) category** |  |  |  |
| --- | --- | --- | --- |
| sarcolemma | GO:0042383 | 8.62e-8 | 9/88 |
| .T-tubule | GO:0030315 | 9.29e-7 | 6/39 |
| cytoskeleton | GO:0005856 | 2.61e-05 | 22/1001 |
| .actin cytoskeleton | GO:0015629 | 4.44e-06 | 9/175 |
| . .striated muscle thin filament | GO:0005865 | 1.65e-9 | 6/13 |
| . .myosin complex | GO:0016459 | 1.22e-7 | 7/42 |
| . . .myosin filament | GO:0032982 | 4.44e-11 | 6/8 |
| cytoplasm | GO:0005737 | 5.77e-05 | 63/5095 |

**Enriched categories among upregulated genes**

| **Biological Process (BP) category** | **ID** | **p-value**  **(adjusted)** | **Number of genes** |
| --- | --- | --- | --- |
| phagocytosis, recognition | GO:0006910 | 4.55e-10 | 6/36 |
| phagocytosis, engulfment | GO:0006911 | 8.57e-10 | 6/48 |
| complement activation, classical pathway | GO:0006958 | 1.08e-9 | 6/44 |
| B cell receptor signaling pathway | GO:0050853 | 1.46e-9 | 6/54 |
| defense response to bacterium | GO:0042742 | 1.41e-8 | 6/87 |
| innate immune response | GO:0045087 | 3.87e-06 | 6/319 |
| immunoglobulin production | GO:0002377 | 3.63e-05 | 3/25 |

| **Molecular Function (MF) category** |  |  |  |
| --- | --- | --- | --- |
| antigen binding | GO:0003823 | 0.00e+00 | 7/37 |
| mmunoglobulin receptor binding | GO:0034987 | 1.4e-10 | 6/34 |

| **Cellular Component (CC) category** |  |  |  |
| --- | --- | --- | --- |
| immunoglobulin complex, circulating | GO:0042571 | 2.22e-10 | 6/28 |
| blood microparticle | GO:0072562 | 1.54e-8 | 6/101 |
| external side of plasma membrane | GO:0009897 | 1.80e-06 | 6/224 |
