# Supplementary material for: Soluble dimeric prion protein ligand activates Adgrg6 receptor but does not rescue early signs of demyelination in PrP-deficient mice
Source: PLoS One. 2020 Nov 12;15(11):e0242137. doi: 10.1371/journal.pone.0242137 (PMC7660510; doi:10.1371/journal.pone.0242137)
Supplement: Attachment — Submitted filename: Response to Reviewers.docx [file pone.0242137.s005.docx]

Revision [PONE-D-20-23889] - [EMID:3f6951fba2cdc223]

**Response to Reviewers and Editor**

Dear Prof. Legname

Thank you for your letter and for the reviewers’ comments concerning our manuscript. The comments are all valuable and helpful for revising our paper. We have studied the comments carefully and made corrections which we hope will meet the reviewers’ concerns. Please find the detailed responses below. Our blot/gel image data are in Supporting Information and we have provided a PDF with all raw images (S1_raw_images).

We are planning to upload our RNAseq data on NCBI geo database. Unfortunately, these servers are currently down. We will provide you with the accession number and respective changes to the data availability statement as soon as possible.

Detailed responses:

1. Please ensure that your manuscript meets PLOS ONE's style requirements, including those for file naming. The PLOS ONE style templates can be found at

https://journals.plos.org/plosone/s/file?id=wjVg/PLOSOne_formatting_sample_main_body.pdf and

https://journals.plos.org/plosone/s/file?id=ba62/PLOSOne_formatting_sample_title_authors_affiliations.pdf

We have formatted the manuscript according to PLOS ONE’s style requirements. We have also used PACE to convert our main figures to the accepted format of PLOS ONE.

2. To comply with PLOS ONE submissions requirements, please provide methods of sacrifice in the Methods section of your manuscript.

We have now provided the details in the Methods section (see p.6 of the revised manuscript with track changes).

3.PLOS ONE now requires that authors provide the original uncropped and unadjusted images underlying all blot or gel results reported in a submission’s figures or Supporting Information files. In your cover letter, please note whether your blot/gel image data are in Supporting Information or posted at a public data repository, provide the repository URL if relevant, and provide specific details as to which raw blot/gel images, if any, are not available. Email us at plosone@plos.org if you have any questions.

We have generated a single pdf file (S1_raw_images) comprising all the uncropped images of blots and gels. The images can be found in Supporting Information, which is also specified in the Methods section of the manuscript.

4. We note that you have stated that you will provide repository information for your data at acceptance. Should your manuscript be accepted for publication, we will hold it until you provide the relevant accession numbers or DOIs necessary to access your data. If you wish to make changes to your Data Availability statement, please describe these changes in your cover letter and we will update your Data Availability statement to reflect the information you provide.

We are planning to upload the RNAseq data on NCBI GEO database. Unfortunately, these servers are currently down. We will provide the accession number and respective data availability statement as soon as possible.

Reviewer #1

I think the authors should be careful with the wording of their conclusions in the Abstract and manuscript title. In the Abstract, it is stated that “no amelioration of the peripheral demyelinating neuropathy was detected.” A more accurate statement may be that “no amelioration of deficits in signaling pathways relevant to myelin maintenance was detected” (or something to that effect). The same goes for the title, since the authors did not actually test whether FT2-Fc “rescues the myelinopathy of PrP-deficient” mice.

We agree with the reviewer that this is a major limitation of our study and we have adapted the wording in title, abstract and discussion of the manuscript to better reflect our findings.

1. Figure 3c clearly shows that Egr2 levels in ZH3 mice are lower than in wild-type mice. However, on page 16 it states in the Figure 3 legend that “ZH3 mice exhibited an age-dependent decrease in Egr2 levels” and in the associated main text that “Egr2 levels progressively decreased when compared to wild-type mice”. In my opinion, the Egr2 Western blot in Figure 3c does not clearly show a progressive decrease in levels with age in either the wild-type or ZH3 mice. The authors should quantify the Egr2 blot signals for these experiments, ideally from ~3 mice per age and genotype.

The reviewer has raised a valid point and it is indeed true that Figure 3c does not support the statement of a “progressive” decrease of Egr2 levels. We have now modified the text accordingly to reflect this in the respective results section and in the figure legend (see p.17). We have refrained from sacrificing more mice to do the quantifications as the precise changes in Erg2 levels do not alter the final conclusions of the experiment and the manuscript. Furthermore, we feel that such data would still not explain why the FT2Fc treatment failed to rescue the molecular signs of neuropathy.

2. For Figure 2b, the authors need to briefly discuss why unexpectedly lower cAMP levels were obtained when using 9 or 10 µM FT2-Fc.

The unexpected shape of the dose-response curve might for example be related to receptor desensitization or to the dimeric nature of FT2Fc. Further studies will need to address the exact activation and desensitization mechanisms of Adgrg6. We included a short discussion about the unexpected dose-response curve in the Results (p.15) and Discussion sections (p.26) of the revised manuscript.

3. In the Methods section on page 5, I think one of the mouse lines should be CAG-/Cre+ (two are listed as CAG-/Cre- at the moment).

We thank the reviewer for highlighting this error. We have now corrected this and another mistake in the Methods section about the breeding of the PrP overexpressing mice, which is now described correctly (p.5).

Reviewer #2

1) The Authors carried out pharmacokinetic studies based on a relatively simple approach: immunochemical detection of FT2Fc in serum. Did they consider using the same approach to measure FT2Fc in nerve tissue? This might be of relevance to determine if FT2Fc failed to elicit a response in vivo simply because it did not attain a sufficient presence in the target tissues.

We were able to detect FT2Fc in nerve lysate by western blotting. We have added a supplementary figure showing the respective blot and included a brief discussion of these results in the manuscript (p.19, p.26).

2) The altered pattern of gene expression in the sciatic nerve induced by FT2Fc treatment was compared with the effect of overexpression of PrP in the tibialis muscle of tg mice. Why not use the pattern of gene expression in the sciatic muscle of these PrP overexpressing mice as a more relevant comparison?

We assume that reviewer #2 meant “sciatic nerve of these PrP overexpressing mice”. The transgenic mice used in our study have only muscle-specific PrP overexpression. Thus, we do not expect significant gene expression changes in the peripheral nerves of these mice. We have extended the discussion about the myotoxicity of PrP-based agents to include the valid criticism raised by reviewer #2 (p.25-26).

3) The Authors suggest that "the dimeric nature of FT2Fc could alter its biological activity when compared to monomeric FT and endogenous PrP" as a possible explanation of its lack of effect (Discussion, line 603). However, the studies conducted in cellula showed that the effect of FT2Fc in activation of the signalling pathways considered was similar to that of monomeric FT.

We agree that FT2Fc works similarly to FT in the two in vitro cellular assays performed here. However, we observed a significantly higher potency of FT2Fc in the cAMP assay as well as an unexpected shape of the dose-response curve when compared FT. These results may point towards an altered biological activity when compared to FT. Furthermore, differences in bioavailability of dimeric proteins (e.g. half-life, avidity) may alter their biologic activity in in vivo experiments. This may be one reason why FT2Fc did not show the expected activity in vivo. We have addressed these concerns in the results and discussion sections.

4) What would be the mechanism by which FT2Fc exerts its PrP overexpression-like effect in gene expression? Would it be mediated by Adgrg6? If so, it means that pharmacological activation of this receptor might be catastrophic if not precisely fine-tuned; if mediated by other receptor, it means that, interestingly, FT has more than one binding partner/function.

Adgrg6 was previously suggested to be expressed in muscle tissue (Moriguchi, Genes Cells, 2004). However, another study (Musa, Ann. N.Y. Acad. Sci., 2019) objected that Adgrg6 detected in several tissues derived from expression in endothelial cells. Thus, we cannot be sure to date whether the detrimental function of PrP in muscle tissue is mediated by Adgrg6 or via other, unknown pathways. We are currently investigating the mechanism of PrP toxicity in skeletal muscle and we will consider both the possibility of Adgrg6-mediated and Adgrg6-independent effects. We have now discussed the questions raised by reviewer #2 in the manuscript (p.25-26).
